# Electricity supply quality and use among rural and peri-urban households and small firms in Nigeria

**DOI:** 10.1038/s41597-023-02185-0

**Published:** 2023-05-12

**Authors:** Setu Pelz, Narges Chinichian, Clara Neyrand, Philipp Blechinger

**Affiliations:** 1 International Institute for Applied Systems Analysis, Laxenburg, Austria; 2 Off-Grid Systems, Reiner Lemoine Institut (RLI), Berlin, Germany; 3 Institute for Theoretical Physics, Technical University of Berlin, Berlin, Germany

**Keywords:** Developing world, Energy access, Climate-change adaptation, Energy supply and demand

## Abstract

We present a household and enterprise energy survey dataset collected within the framework of the PeopleSuN project in Nigeria in 2021. Across three Nigerian geopolitical zones, a total of 3,599 households and 1,122 small and medium-sized enterprises were surveyed. The sample is designed to be representative of rural and peri-urban grid-electrified regions of each zone. Our surveys collect data on demographic and socioeconomic characteristics, energy access and supply quality, electrical appliance ownership and usage time, cooking solutions, energy related capabilities, and supply preferences. We encourage academic use of the data presented and suggest three avenues of further research: (1) modelling appliance ownership likelihoods, electricity consumption levels and energy service needs in un-electrified regions; (2) identifying supply-side and demand-side solutions to address high usage of diesel generators; (3) exploring broader issues of multi-dimensional energy access, access to decent living standards and climate vulnerability.

## Background & Summary

Rural and peri-urban populations in Nigeria continue to suffer unreliable and expensive energy supply. According to the World Bank, the electricity access rate in Nigeria stood at 55.4% in 2020 with a big gap between urban and rural areas (83.9% vs. 24.6%)^1^. At the same time, nearly 30 million Nigerian households depend on wood as a source of cooking fuel, the collection of which is time consuming and mainly done by women^2^. Where there is supply, it is typically unreliable and frequently interrupted by blackouts. The Nigeria Enterprise Survey from the World Bank showed that 27% of Nigerian firms identified reliability of electricity supply as the main obstacle to their business^3^. On average, 32.8 power outages were reported to occur in a typical month leading to an estimated 11% loss in sales value^3^. The average grid-connected household receives just 6.6 hours of supply on a typical day, linked to a per capita consumption of just 144kWh per year^4^. In comparison, the annual per capita consumption in Ghana and South Africa is respectively 351 kWh and 4,198 kWh. Plagued by issues of supply quality, many Nigerians have resorted to self-generation using petrol and diesel generators, spending approximately 1.56 trillion Naira (3.76 billion USD, using an average exchange rate in 2021) per year on fuel^5^.

While global efforts are accelerating under the banner of achieving Sustainable Development Goal 7 (SDG7) by 2030, progress in Nigeria remains hindered by limited data availability, among other barriers. Data describing the energy access deficit in Nigeria exists (see Table 1), however, there is limited disaggregate information describing the supply quality in the existing network and the unmet demand in ‘un-electrified’ regions. In this data descriptor, we present primary survey data collected to fill this and other gaps through the ‘People Power: Optimizing off-grid electricity supply systems in Nigeria’ project (PeopleSuN)’^6^. PeopleSuN is funded by the German Federal Ministry of Education and Research (BMBF) within the funding initiative ‘Client II - International Partnerships for Sustainable Innovations. Data collection followed extensive stakeholder discussions in Nigeria under the PeopleSuN project to define the data gap and the necessary survey and sampling strategy to address this. The questionnaires used draw from specific modules within established surveys capturing energy-related data, most directly from the Multi-Tier Framework for Measuring Energy Access surveys^7^. The surveys provide data on household and enterprise characteristics, energy supply and consumption. They also capture preferences, trust in institutions and several gender-disaggregated variables. Our sample is representative of grid-electrified rural and peri-urban regions across three geopolitical zones with large energy access deficits. This data provides important insight into actual energy supply quality and use among ‘electrified’ communities and can be used to improve models of energy demand in similar but currently ‘un-electrified’ communities. The final sample includes 3,599 households and 1,122 small and medium sized enterprises from 225 enumeration areas across the three geopolitical zones.

**Table 1:** Overview of existing sources of data describing energy access and use in rural and peri-urban Nigeria.

Data source	Description	Year	Representativeness	Energy data
General Household Survey/Living Standards Measurement Study (World Bank) https://www.worldbank.org/en/programs/lsms	National household living standards survey.	Multiple survey rounds (most recent 2018)	Nationally representative survey of approximately 5,000 households, which are also representative of the six geopolitical zones.	Household socioeconomics, assets and general energy access questions.
Nigeria SE4ALL Platform. Federal Ministry of Power, Nigeria. https://nigeriase4all.gov.ng/	Platform provides access to geospatial data and market data related to grid-connected and off-grid energy in Nigeria.	Frequently updated.	Geospatial data for all of Nigeria.	Spatial data describing locations of consumers, existing and planned grid and off-grid energy infrastructure, and administrative boundaries.
Energy Sector Management Assistance Program (ESMAP), World Bank. Nigeria - Multi-Tier Framework for Measuring Energy Access Household Survey (MTF) 2018. https://microdata.worldbank.org/index.php/catalog/3865	The MTF survey assesses energy access and use in North, North-West Nigeria.	2018	Stratified random sampling, with equal allocation between urban and rural areas and equal allocation between grid-user and non grid-user households in North, North-West of Nigeria.	Data on energy access, types of energy sources used, appliance ownership and energy expenditure.
Falchetta, G., Pachauri, S., Parkinson, S. *et al*. A high-resolution gridded dataset to assess electrification in sub-Saharan Africa. Sci Data 6, 110 (2019). 10.1038/s41597-019-0122-6	A high-resolution gridded dataset to assess electrification in sub-Saharan Africa	2019	sub-Saharan Africa	Spatial distribution and temporal evolution of electricity access.
DHS surveys - Nigeria https://dhsprogram.com/	Demographic and Health Surveys collect data on population, health, and nutrition in Nigeria.	Multiple survey rounds (most recent 2018)	Nationally representative, with data available for individual states and regions.	Data on household electricity access, types of cooking fuels, and ownership of appliances.
n/a	Enterprise surveys provide data on the business environment, including energy-related aspects, through interviews with firm managers and owners.	Multiple survey rounds (most recent in 2014)	Covers various regions in Nigeria with a focus on the formal sector.	Energy reliability, power outages, and energy expenditures for businesses.

## Methods

**Fig. 1:**
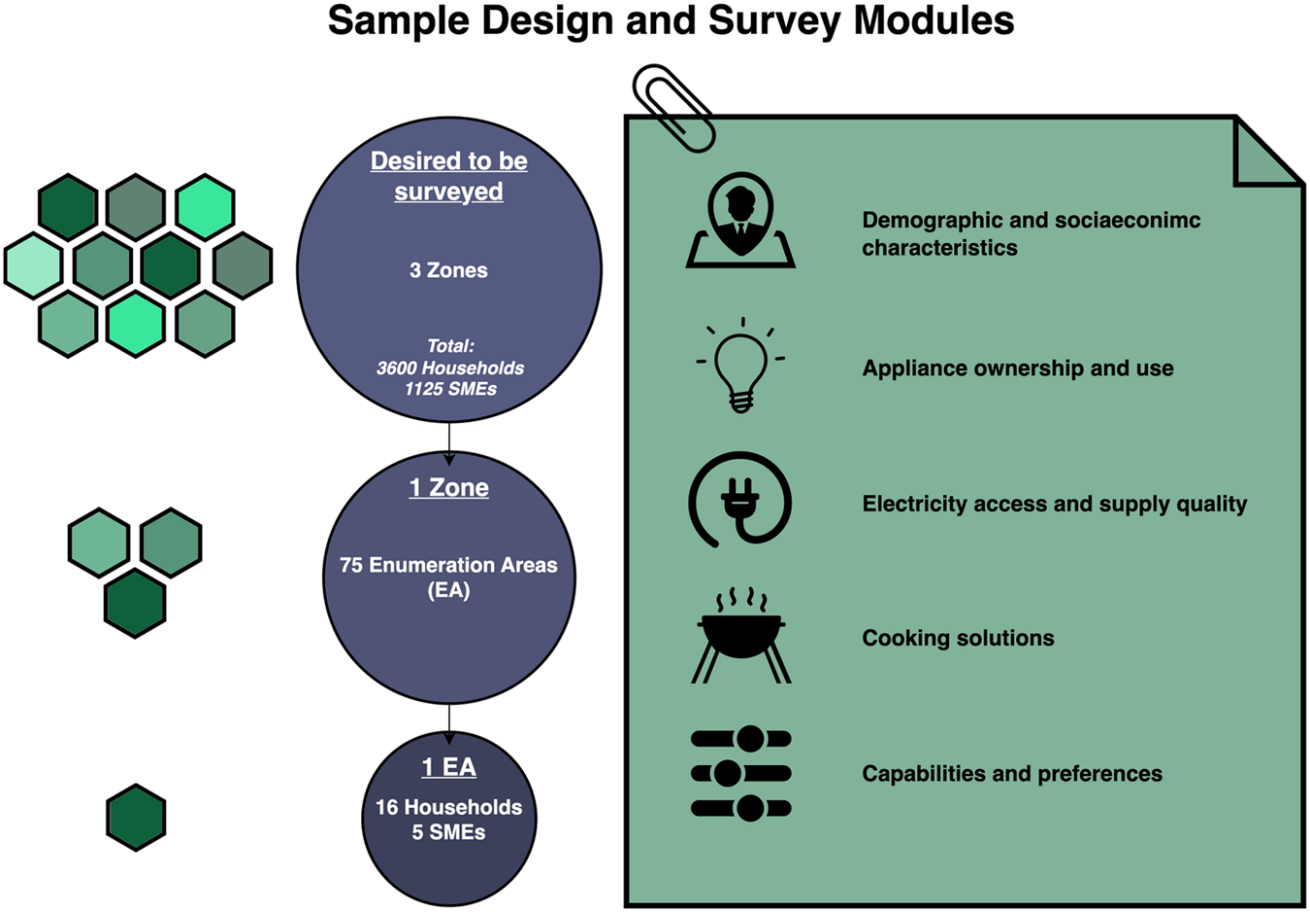
Sample design and questionnaire modules. Three of six Nigerian zones, North West, North Central and South South were selected. These zones were chosen based on lower urbanisation rates (excluding the South West and South East) and safety and logistical considerations (excluding North East). Seventy-five enumeration areas (EA) were marked in each zone [total of 225 EAs]. Within each enumeration area, sixteen households and five enterprises were planned to be surveyed. The desired distribution of EAs and the final implementation are shown respectively in Table 4. The surveys cover a wide range of questions regarding demographic and socioeconomic variables, electricity availability and supply quality, appliance ownership and use, available cooking solutions, and capabilities and preferences.

Figure 1 provides an overview of the sample design and survey modules used. Complete codebooks describing the questionnaires are available here: https://dataverse.harvard.edu/dataset.xhtml?persistentId=doi:10.7910/DVN/GTNEJD^6^. We now describe the data collection methods in detail.

### Sample frame

**Fig. 2:**
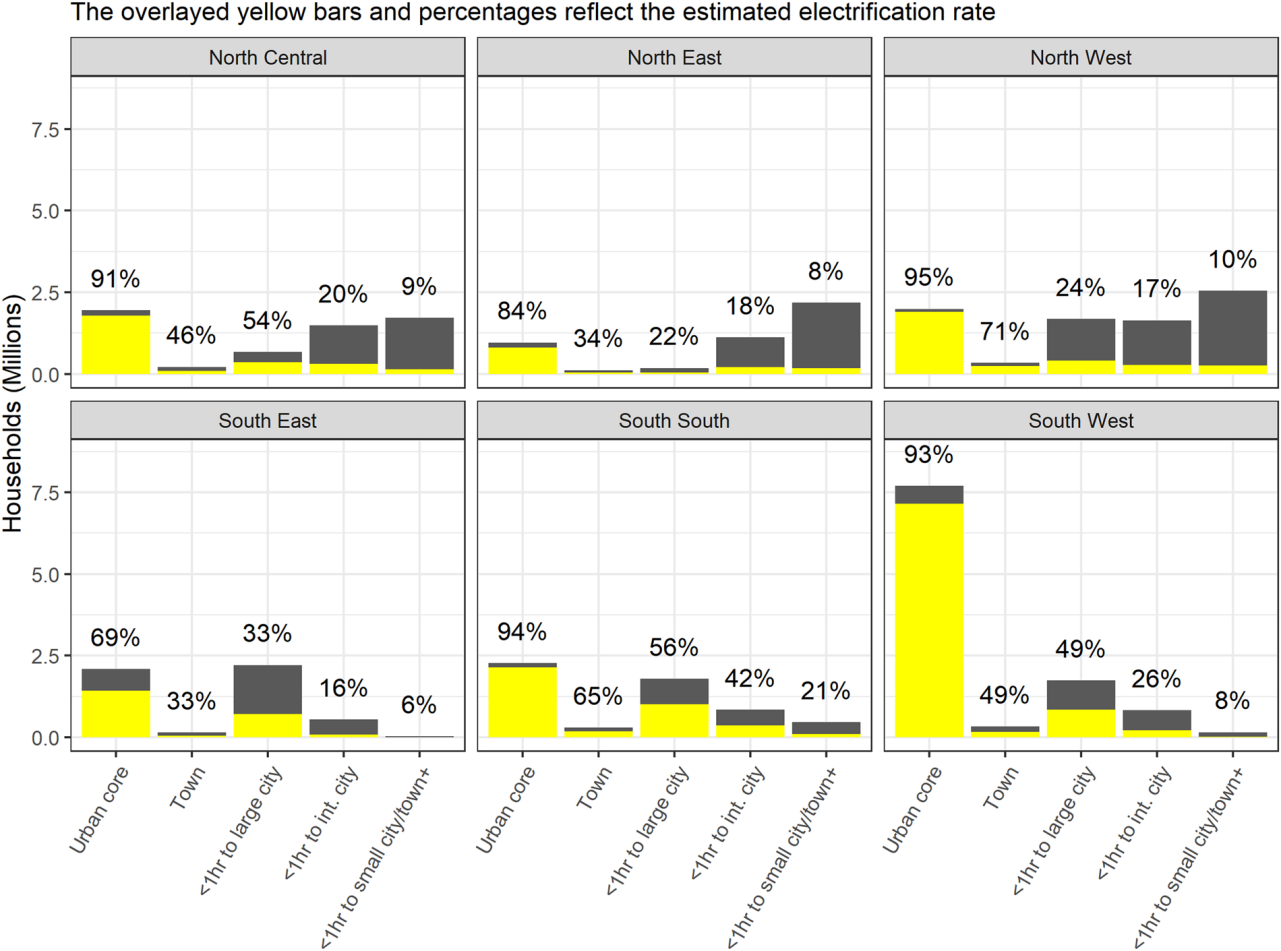
Distribution of households and share electrified by rurality across Nigeria. Population estimates are taken from the latest bottom-up WorldPop dataset for Nigeria (V1.2, see^9^). These were converted to household estimates using the average household size in each state as captured by the 2018 DHS survey (see^10^). Rurality is defined using an aggregation of the URCA definition (see^8^). Each group reflects a more rural region, starting with cities of all sizes which are aggregated to the group ‘Urban core’, to very remote areas including those further than 1 hour from a small city or town which are aggregated into the final group ‘<1 hr to small city/town+’. Electrification estimates are taken from AtlasAI spatial electrification data for the year 2020 (see atlasai.com). Our work focusses on grid-electrified households outside the urban core.

Three geopolitical zones were selected for sampling: North West, North Central, and South South. These zones were purposefully chosen based on lower rates of urbanization (excluding South West and South East) and safety and associated logistical concerns (excluding North East). The sample frame was then developed using remotely sensed datasets due to the lack of up-to-date administrative data. Four remotely sensed spatial datasets were used, as shown in Table 2. The focus of this work was on understanding energy supply quality and use among grid-electrified households living in rural and peri-urban regions (outside urban centers). Urbanization was defined using the ‘Urban Rural Catchment Area’ dataset (URCA), which stratified the population by travel time to the nearest agglomeration. Further detail on the URCA sub-categories and their definition can be found in^8^. For the purposes of this work, the URCA categories were aggregated into an urban core and varying degrees of rurality, as shown in Table 3. Figure 2 describes the distribution of households across these categories and separated into those assumed to be electrified as per the ELEC dataset. All Towns (>0.02 M and <0.05 M population) and regions that were within or further than 1 hour travel time from a city boundary were defined as outside the urban core and relevant to this study. Estimated population sizes and electrification rates within each aggregate spatial category across all six geopolitical zones are shown in Fig. 2, providing further intuition regarding the selection of North Central, North West, and South South as zones of study.

**Table 2:** Overview of remotely sensed datasets used in defining the sample frame.

Acronym	Detail	Resolution/Year	Source
(WPOP)	WorldPop Bottom-up Population Estimate	0.1 km^2^, 2020	^9^
(URCA)	Urban-Rural Catchment Area	1 km^2^, 2015	^8^
(ELEC)	AtlasAI Electrification Data	4 km^2^, 2020	(see atlasai.com)
(ASSET)	AtlasAI Asset Index	4 km^2^, 2020	(see atlasai.com)

**Table 3:** Original URCA spatial categories and corresponding PeopleSuN aggregated categories.

	(a) URCA original categories						
	Large cities		Intermediate cities		Small cities		Towns
	>5 M	1–5 M	0.5–1 M	0.25–0.5 M	0.1–0.25 M	0.05–0.1 M	0.02–0.05 M
Urban centers	1	2	3	4	5	6	7
Peri-urban 0–1 hours	8	9	10	11	12	13	20a
Peri-rural 1–2 hours	14	15	16	17	18	19	20b
2–3 hours	21	22	23	24	25	26	27
Hinterlands >3 hours							28/29
	**(b) URCA PeopleSuN aggregated categories**						
Urban centers	Urban Core						Cat 1
Peri-urban 0–1 hours	Cat 2		Cat 3		Cat 4		
Peri-rural 1–2 hours				Cat 4			
2–3 hours				Cat 4			
Hinterlands >3 hours							Cat 4

URCA categories reflect distinct catchment areas describing distance from urban agglomerations taking into account road quality among other factors. These are aggregated for the purposes of the PeopleSuN sample. See^8^ for further detail regarding the categorisation approach.

### Sampling

In total 3,600 households were intended to be surveyed, split evenly into the three geopolitical zones. Households were sampled from 225 enumeration areas (EAs) consisting of 16 households each. In addition, 5 firms were to be surveyed in each enumeration area, giving a total sample of 1,125 firms. Within each zone, half of the states (four in total per state) were selected using systematic probability proportionate to size sampling (PPS) in order to reduce survey costs. The count of households at the state level was used as the weighting variable in the PPS sampling. This approximates a representative zonal sample when not able to visit all states within each zone. The state-level pre-sampling may introduce bias with respect to the rurality of sparsely populated states not included in our sample. To address this the distribution of households with respect to the URCA PeopleSuN categories across *all* states in each zone is used to adjust subsequent sampling within selected states. Table 4 describes the approximate proportion of grid-connected households in each zone and category. This is done including all states, not just those sampled. The proportions therefore represent the share of people living in each category that should be replicated within the restricted sample of states. This proportional stratification results in a sample size within each zone and category that reflects, as far as possible, a representative sample of grid-electrified rural and peri-urban households across the zone.

**Table 4:** Desired and final enumeration areas per zone and URCA PeopleSuN category.

Zone	URCA PeopleSuN category	Households	Share	Desired EAs	Sampled EAs	Grid-connected
North Central	Town (0.02 M)	98297	0.11	8	9	9
North Central	<1 hr to large city	364067	0.4	30	30	30
North Central	<1 hr to int. city	301774	0.33	25	25	24
North Central	<1 hr to small city/town+	147266	0.16	12	11	11
North West	Town (0.02 M)	243730	0.2	15	17	17
North West	<1 hr to large city	413041	0.35	26	27	26
North West	<1 hr to int. city	275368	0.23	17	18	18
North West	<1 hr to small city/town+	261740	0.22	17	13	8
South South	Town (0.02 M)	188965	0.11	8	10	10
South South	<1 hr to large city	1012942	0.61	46	46	46
South South	<1 hr to int. city	356610	0.22	17	16	16
South South	<1 hr to small city/town+	98675	0.06	5	3	3

Households describes the approximate number of grid-connected households in each category. Desired EAs describes the desired allocation of enumeration areas providing a representative sample of rural and peri-urban grid-connected households in each zone. Sampled EAs describes the actual final sample following implementation and Grid-connected describes those that were indeed grid-connected.

The final enumeration areas within each zone are selected using PPS sampling once again, weighted by the total number of households in each cell to achieve the sample size by zone as defined in the Desired EAs column. A total sample of 225 enumeration areas is equivalent to a zonal sample of 75 enumeration areas, distributed as per the proportional stratification across each of the three zones. Within each zone, this results in a sample that is representative of grid-electrified households outside the urban core while reducing the cost of survey implementation. Analysis can therefore be conducted at the zonal level as is, and re-weighted to arrive at a representative ‘national’ sample across all three contiguous zones if desired. The necessary weights for aggregate analyses across all three contiguous zones are provided with the dataset.

### Implementation

Survey implementation was conducted by eHealth Africa (eHA), who also secured state-government permission to conduct surveys in each state. The survey was conducted using the Kobo Collect Computer-Assisted Personal Interviews (CAPI) technology. All EAs within security compromised Local Government Areas (LGAs) were identified and shared with the research team for replacement. From the total of 255 EAs originally planned (reflecting a desired sample of 225 EAs and 30 buffer EAs), 247 EAs remained during implementation. From the 21st to the 23rd June 2021, a training of trainers was conducted by the research team for eHA’s key staff and field supervisors who were scheduled to work on the project. Following this, the translation of survey instruments into local languages (Hausa and Pidgin) was finalized on the 12th of July. This was followed by field testing which was conducted on the 13th and 14th of July 2021, and a field testing report was submitted on 16th July 2021. Step-down training of enumerators was simultaneously conducted between 25th - 29th July 2021 in three locations (Kano, Abuja, and Akwa Ibom) to accommodate the enumerators by zone and reduce travel costs. Kano hosted the North West states’ enumerators while Abuja and Akwa-Ibom hosted North Central and South-South enumerators respectively with the report submitted on the 30th of July 2021. Pre- and post-training assessments were conducted and enumerators that met the evaluation benchmarks were finally selected. A total of 60 enumerators and 12 state supervisors were engaged in the quantitative survey activity.

**Fig. 3:**
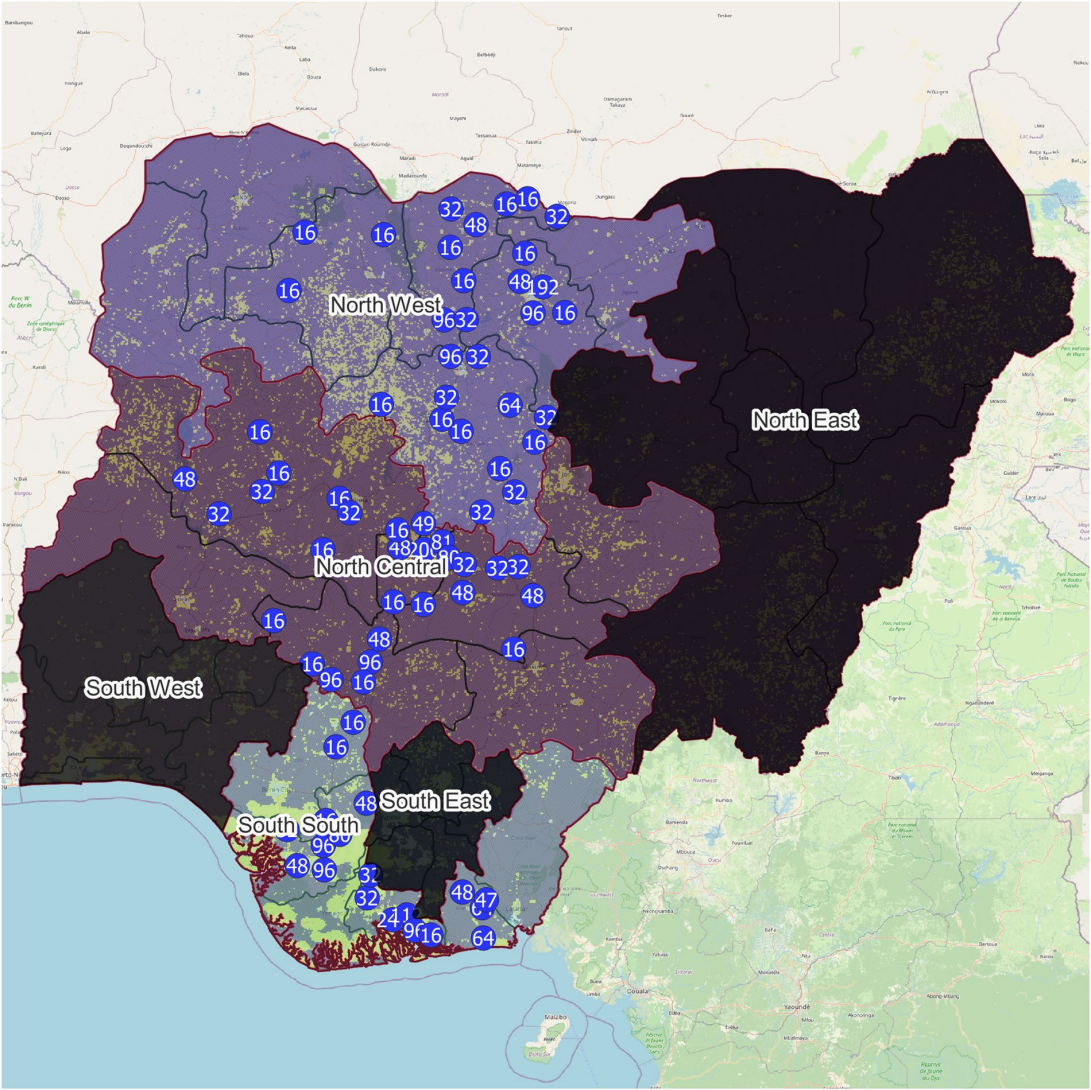
Visualisation of the spatial distribution of the household sample. Geopolitical zones that were not sampled are shaded dark grey. Actual sample locations have been jittered up to 10 kilometres and labels reflect the total number of households sampled (numbers greater than 16 indicate a cluster of enumeration areas). The basemap includes the spatial electrification coverage for 2020 as estimated by AtlasAI, shown as yellow pixels. Any geographic boundary inaccuracies are unintentional. All secondary data is from identical sources as stated for Fig. 2.

Data collection commenced on August 7th. A team comprising two enumerators (mostly paired male and female) worked to cover 16 households and 5 enterprises in each EA. Community leaders and local authorities were consulted before enumerators’ visits and commencement of activity at the LGA and community levels. The team faced challenges in gathering survey data due to security, communal clashes and inaccessibility in certain EAs, requiring replacement with a list of buffer enumerations areas within each zone. As a result, there is some small level of bias with respect to the zones given the mismatch in URCA group proportions, which is shown in Table 4. This remains small overall and the mechanism underlying this is transparently communicated here. Furthermore, one enumeration area in North Central, and six in North West were not grid-connected (despite being identified as electrified within the AtlasAI data), but were using electricity in other forms such as diesel generators or solar devices. One EA (AK12) contains a sample of 15, rather than 16 households which was not able to be completed due to banditry on the final day of enumeration, giving us a final sample of 3,599 households. Figure 3 depicts the full sample of enumeration areas with household counts shown as labels. Points have been jittered up to 10 km.

## Data Records

All data is publicly available here https://dataverse.harvard.edu/dataset.xhtml?persistentId=doi:10.7910/DVN/GTNEJD^6^.

## Technical Validation

Prior to data collection, a thorough preparatory program was implemented, starting with a training of trainers session conducted by the research team for eHA’s key staff and field supervisors from June 21st to 23rd, 2021. The sessions aimed to establish a strong foundation in survey methodology and the specific techniques required for this project. Following the training of trainers, field testing was conducted on July 13th and 14th to identify potential issues or inconsistencies in the survey tool or the Hausa and Pidgin translations. A field testing report was submitted on July 16th, providing valuable insights to improve the survey instruments and enumerator guidelines. Subsequently, step-down training of enumerators was carried out between July 25th and 29th, 2021, in three locations (Kano, Abuja, and Akwa Ibom) to accommodate enumerators by zone and reduce travel costs. The training focused on data collection techniques, handling of survey instruments, and ethics in data collection. Pre- and post-training assessments were conducted, and enumerators meeting the evaluation benchmarks were finally selected. In total, 60 enumerators and 12 state supervisors were engaged for the quantitative survey activity.

Data collection finally commenced on August 7th, 2021, with teams of two enumerators covering 16 households and five enterprises in each enumeration area (EA). Community leaders and local authorities were consulted before the enumerators’ visits and commencement of activity at the LGA and community levels. During data collection, standard outlier and consistency checks were performed daily across the dataset. This process involved running outlier detection algorithms and cross-validating the collected data for accuracy and completeness. Inconsistencies or outliers identified were either approved, rejected, or flagged for clarification. This real-time validation process helped maintain the quality of the data collected and minimized the likelihood of errors. Security challenges, communal clashes, and inaccessibility in certain EAs necessitated the use of buffer enumeration areas, resulting in a small level of bias with respect to the zones. This bias, however, remains minimal, and the underlying mechanism is transparently communicated.

Following data collection, a three-week data cleaning process was initiated, which involved extensive exchange with the enumerators and supervisors. This process allowed for the identification and rectification of any remaining inconsistencies, errors, or omissions in the dataset. Enumerators were consulted for clarification and verification of flagged data points, ensuring that the final dataset was both accurate and reliable, and any issues transparently reported. For example, during the cleaning process, it was found that one EA in North Central and six in North West were not grid-connected but used other forms of electricity like diesel generators or solar devices. Additionally, one EA (AK12) contained a sample of 15 instead of 16 households due to banditry on the final day of enumeration, resulting in a final sample of 3,599 households.

## Usage Notes

We present here a novel survey dataset describing demographic and socioeconomic characteristics, electricity access and supply quality, electrical appliance ownership and usage time, cooking solutions, capabilities, and preferences across households and enterprises living in grid-electrified communities outside the urban core in Nigeria. The dataset is provided as a set of .csv and .xlsx files. Table 5 describes each file.

**Table 5:** Overview of files included in the presented dataset.

Filename	Description	Usage
peoplesun_hh_odk_codebook.xlsx	Household questionnaire codebook	Link to household dataset by variable name
peoplesun_hh_odk_choices.xlsx	Household multi- or single-select question labels	Link to codebook by list name
peoplesun_hh_anon.csv	Anonymised household survey dataset	Apply weights when aggregating zones
peoplesun_hhstoves_anon.csv	Household stove roster	Link to household dataset by household ID
peoplesun_hhapps_anon.csv	Household appliance roster	Link to household dataset by household ID
peoplesun_ent_odk_codebook.xlsx	Enterprise questionnaire codebook	Link to enterprise dataset by variable name
peoplesun_ent_odk_choices.xlsx	Enterprise multi- or single-select question labels	Link to codebook by list name
peoplesun_ent_anon.csv	Anonymised enterprise survey dataset	Apply weights when aggregating zones
peoplesun_entstoves_anon.csv	Enterprise stove roster	Link to enterprise dataset by enterprise ID
peoplesun_entapps_anon.csv	Enterprise appliance roster	Link to enterprise dataset by enterprise ID
peoplesun_entequips_anon.csv	Enterprise equipment roster	Link to enterprise dataset by enterprise ID
eaidgeokey_anon.csv	Basic geospatial data per enumeration area	Link to datasets by enumeration area ID

This dataset can inform decision makers, project developers and researchers about the current energy supply situation and model future energy use towards securing a decent living standard for all in rural and peri-urban areas of Nigeria. Our preliminary assessment points to three key areas of further research using this data. First, we propose *modelling appliance ownership likelihoods, electricity consumption levels and energy service needs in un-electrified regions*. Surveyed use of appliances among grid-connected communities can serve as a proxy to estimate future electricity demand for similar households which are currently not grid connected. Second, we propose *identifying supply-side and demand-side solutions to address high usage of diesel generators*. The collected data provides evidence of grid supply reliability and backup prevalence solutions among rural and peri-urban grid-electrified communities, as well as energy service needs. Thirdly, we propose *exploring broader issues of multi-dimensional energy access, access to decent living standards and climate vulnerability*. The data captures objective appliance ownership and use as well as subjective satisfaction with access to basic energy services such as keeping oneself at a comfortable temperature. Similarly, with regard to productive uses of electricity, the data describes appliance and equipment ownership and use, as well as subjective satisfaction with energy supply affordability, reliability and adequacy.

A common theme across these three research areas is the importance of increasing the central grid supply quality and in providing cost-competitive decentralized alternatives to improve living conditions and business perspectives in Nigeria. Although our data descriptor focusses on energy access, we understand that achieving SDG7 - providing sustainable energy for all - is not the silver bullet for improving living conditions and business perspectives on its own. A myriad of accompanying factors such as access to other basic services including education, sanitation and health, access to capital and broader issues of gender equality must be addressed. Nevertheless, SDG7 is a key enabling achievement as many other SDGs depend upon sustainable energy access. It is clear that achieving improving energy access in Nigeria is both about new connections and about increasing the supply quality in weak grid areas. The existing grid network needs to be strengthened and decentralised renewables will play a role in both peri-urban and rural regions. Furthermore, in order to satisfy energy service needs in the context of a changing global climate, demand-side policies supporting household acquisition of appliances necessary for a decent living standard must be explored. A cross-cutting analysis combining expertise from institutions responsible for centralised network planning and those responsible for decentralised electrification efforts would be ideal to strategically plan these efforts. We hope that the data we present can be useful in providing evidence for such an analysis.

## Data Availability

As we are presenting collected survey data, no custom code was used or is necessary to generate or work with this data. Complete codebooks describing the questionnaires are available here: https://dataverse.harvard.edu/dataset.xhtml?persistentId=doi:10.7910/DVN/GTNEJD^6^.
