# Author Correction: Electricity supply quality and use among rural and peri-urban households and small firms in Nigeria

**DOI:** 10.1038/s41597-023-02771-2

**Published:** 2023-12-04

**Authors:** Setu Pelz, Narges Chinichian, Clara Neyrand, Philipp Blechinger

**Affiliations:** 1 International Institute for Applied Systems Analysis, Laxenburg, Austria; 2 Off-Grid Systems, Reiner Lemoine Institut (RLI), Berlin, Germany; 3 Institute for Theoretical Physics, Technical University of Berlin, Berlin, Germany

**Keywords:** Developing world, Energy access, Climate-change adaptation, Energy supply and demand, Developing world, Energy access, Climate-change adaptation, Energy supply and demand

Correction to: *Scientific Data* 10.1038/s41597-023-02185-0, published online 12 May 2023

The Acknowledgements section of article contained a spelling error for the name of one contributor to the work. This has now been corrected in the pdf and HTML versions of the article.

